# PSW-Designer: An Open-Source Computational Platform for the Design and Virtual Screening of Photopharmacological Ligands

**DOI:** 10.1021/acs.jcim.3c01050

**Published:** 2023-10-13

**Authors:** Icaro A. Simon, Evert J. Homan, Maikel Wijtmans, Michael Sundström, Rob Leurs, Iwan J. P. De Esch, Barbara A. Zarzycka

**Affiliations:** † Division of Medicinal Chemistry, Faculty of Science, Amsterdam Institute for Molecular and Life Sciences, Vrije Universiteit Amsterdam, 1081 HZ Amsterdam, The Netherlands; ‡ Science for Life Laboratory, Department of Oncology-Pathology, Karolinska Institutet, S-171 76 Stockholm, Sweden; § Centre for Molecular Medicine, Karolinska Institutet, S-171 76 Stockholm, Sweden

## Abstract

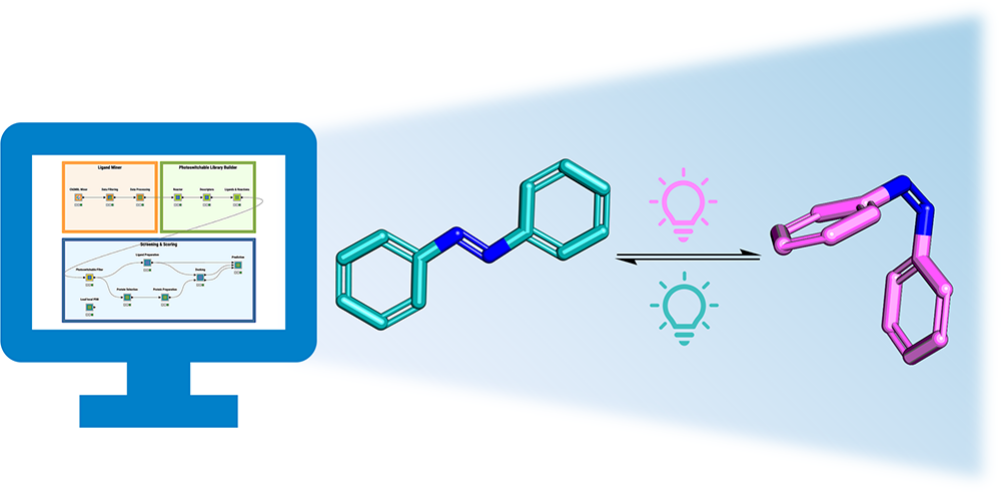

Photoswitchable (PSW) molecules offer an attractive opportunity for the optical control of biological processes. However, the successful design of such compounds remains a challenging multioptimization endeavor, resulting in several biological target classes still relatively poorly explored by photoswitchable ligands, as is the case for G protein-coupled receptors (GPCRs). Here, we present the PSW-Designer, a fully open-source computational platform, implemented in the KNIME Analytics Platform, to design and virtually screen novel photoswitchable ligands for photopharmacological applications based on privileged scaffolds. We demonstrate the applicability of the PSW-Designer to GPCRs and assess its predictive capabilities via two retrospective case studies. Furthermore, by leveraging bioactivity information on known ligands, typical and atypical strategies for photoswitchable group incorporation, and the increasingly structural information available for biological targets, the PSW-Design will facilitate the design of novel photoswitchable molecules with improved photopharmacological properties and increased binding affinity shifts upon illumination for GPCRs and many other protein targets.

## Introduction

Photopharmacology is a relatively incipient branch of pharmacology that uses light to modulate biologically active ligands.^1^ It enables the noninvasive modulation of biological targets with high temporal and spatial resolution, thus allowing for the “remote control” of binding and signaling mechanisms in near real-time and with minimal disturbance to the native environment.^2^ This light-induced control is achieved by hindering the action of a molecule via the introduction of a photoresponsive group that can either block the recognition of an active compound by its biological target (*photocaging*)^3^ or lead to a configurational change that alters or disrupts the interaction of one of the photoisomers of the molecule with its target (*photoswitching*).^2,4^

**Figure 1:**
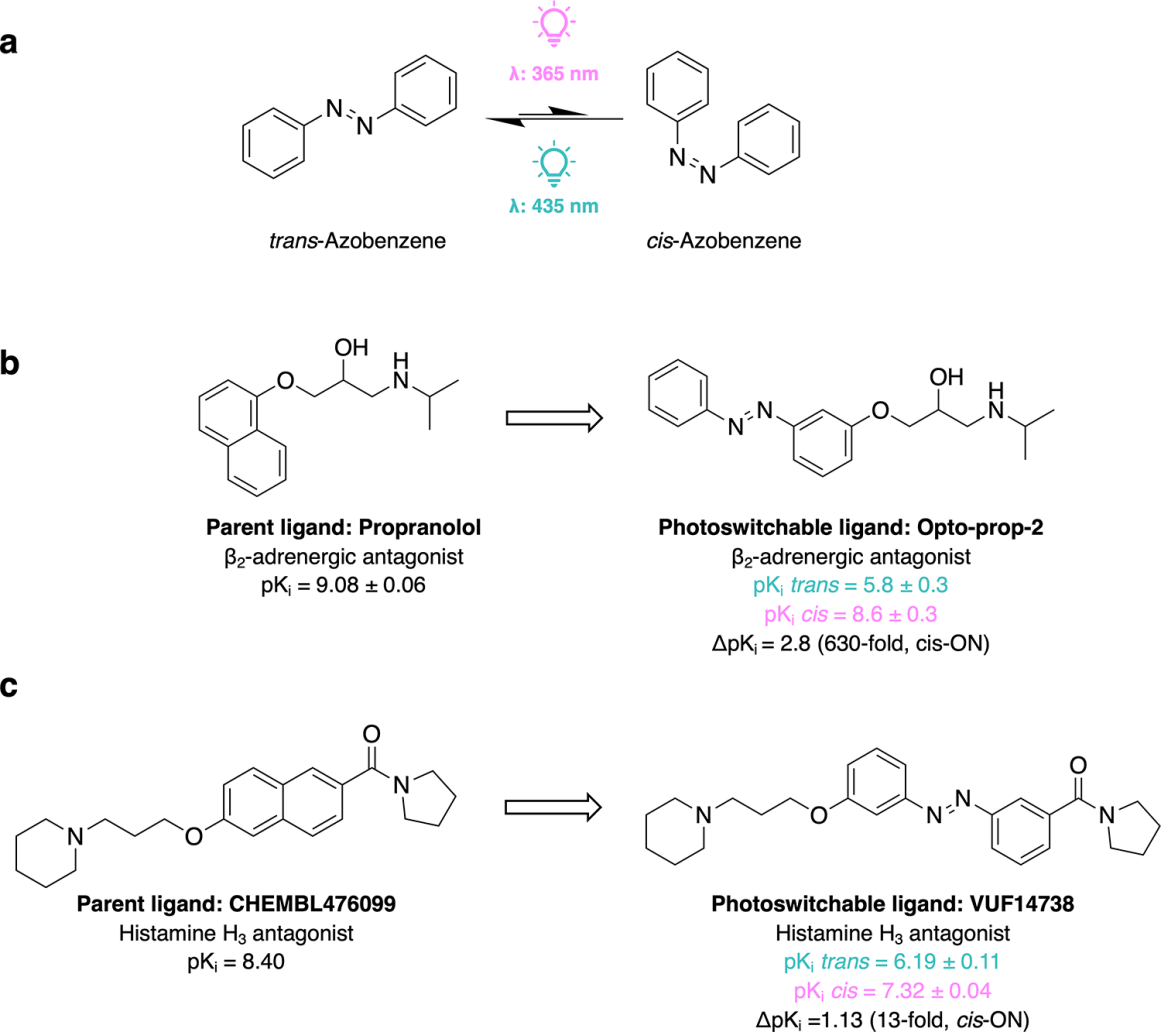
(a) The reversible photoisomerization of azobenzene from the thermally stable *trans* to the *cis* configuration. (b, c) The parent compounds and their successful photoswitchable antagonists for β_2_-adrenergic (b) and histamine H_3_ (c) G protein-coupled receptors.

Photoswitching relies on the reversible photoisomerization of molecules upon illumination at an appropriate wavelength—often *trans–cis* (E/Z) isomerism—leading to configurational isomers with contrasting structural, physicochemical, and electronic properties.^4,5^ The reversibility of this process allows for the activation and deactivation of the molecule upon illumination at distinct wavelengths and/or thermal relaxation of the less stable photoisomer to the more stable one, thereby offering unprecedented opportunities to control target response while potentially reducing *off*-target effects.^5^ A classic example of a photoresponsive group is diphenyldiazene (azobenzene), which reversibly isomerizes from the thermally stable *trans* to the metastable *cis* configuration (Figure 1a) upon illumination at λ ≈ 365 nm (ultraviolet light).^6^ The reverse process occurs spontaneously through thermal relaxation but can also be achieved with illumination at λ ≈ 435 nm (blue visible light).^6^

By merging protein recognition (i.e., pharmacophore) elements to this light-responsive moiety, photoswitchable (PSW) molecules can be designed in which one of the isomers will not bind, bind weakly, or elicit a distinct response on the biological target than its counterpart, hence allowing the photomodulation of binding affinity, potency, and/or efficacy.^7−10^ For example, a *cis*-ON ligand is a molecule that is less active in the *trans*-state and acts only on the biological target upon illumination and photoconversion to the metastable *cis-*state. In practice, the design of such photoswitchable molecules is challenging, as it involves a multiobjective optimization that encompasses the simultaneous adjustment of pharmacodynamics (e.g., target binding affinity and isomeric differences/shifts), pharmacokinetics (e.g., aqueous solubility and/or membrane or BBB penetrability), and photochemical characteristics (isomerization wavelengths, sufficient conversion at the photostationary state, relaxation half-life, etc.), properties that sometimes oppose each other.^11^

A common strategy in the design of photoswitchable ligands is to modify the chemical scaffold of known drugs or tool compounds with well-established bioactivity profiles by incorporating the photoresponsive group, rather than by *de novo* design.^8^ This incorporation can be performed in the core of a suitable parent molecule (*azologization*) or its periphery (*azoextension*).^12^ Azologization can lead to enhanced isomeric shifts by promoting large changes in ligand regions that are critical for its recognition by the target. However, there is a limited scope of parent ligands bearing functional groups (named *azologs* or *azosters*) that can be replaced *a priori* by an azo bond, azobenzene, or another photoisomerizable group. Unfortunately, this incorporation can lead to a significant decrease in binding affinity and/or bioactivity compared to the parent compound.^12,13^ Azoextensions, on the other hand, are capable of maintaining the bioactivity of the parent ligand, as an azobenzene or other photoisomerizable group is incorporated onto a terminal region of the parent molecule (*full-azoextension*) or a single phenyldiazene is incorporated into a suitable position of an existing aryl group (*half azoextension*), which becomes part of the photoresponsive group.^8^ These strategies, however, can result in a lack of distinction between isomers, as the photoresponsive groups are frequently attached to distal and solvent-exposed regions on the parent molecule, with neglectable interaction with the biological target in both E/Z configurations.^14,15^ All of these challenges complicate the development of novel photoswitchable molecules.

An illustrative example of this difficulty is the development of novel photoswitchable ligands for G protein-coupled receptors (GPCRs), which despite being some of the most prominent drug targets, have been relatively poorly explored in photopharmacology until now.^16,17^ A recent review^11^ showed that less than 5% of the ∼300 nonolfactory class A GPCRs have been targeted by photoswitchable ligands so far, and most of the reported ligands display little distinction in pharmacological properties between the isomers (small isomeric shifts) and often with a significant reduction in binding affinity in comparison to the parent ligands.^11,17^ GPCR photopharmacology would greatly benefit from the precise and modulatory control offered by better photoswitchable ligands.^18,19^

In this study, we report a computational platform (the PSW-Designer) for the design and structure-based virtual screening of photoswitchable ligands. By retrieving and processing the structural and bioactivity data of known ligands, followed by the incorporation of the photoswitchable (azobenzene) group into these parent molecules through both azologization and azoextension strategies, a library of novel photoswitchable compounds can be generated and computationally assessed for complementarity to protein binding sites. We focus our efforts on the azobenzene moiety as this is by far the most commonly used photoswitchable motif in photopharmacology.^11,20^ The virtual screening strategy takes into consideration relevant information on the parent molecule to assign the active and less active photoswitchable isomers and to estimate the binding affinity shifts upon isomerization, thus helping in hypothesis generation and ligand prioritization for synthesis. While we focus on GPCRs (namely, β_2_-adrenergic and histamine H_3_ receptors) to demonstrate the utility of the PSW-Designer, this platform can be applied to any biological target with bioactivity data available in ChEMBL.^21^

## Methods

The PSW-Designer is implemented in KNIME Analytics Platform^22^ (version 4.7.3), an open-source data management pipeline editor in which distinct operations are performed in data table objects via “nodes”, where the output of a node becomes the input for the following. These nodes can be combined into metanodes and components, which allow aggregate operations and user interaction with the data processing and analysis steps.

**Figure 2:**
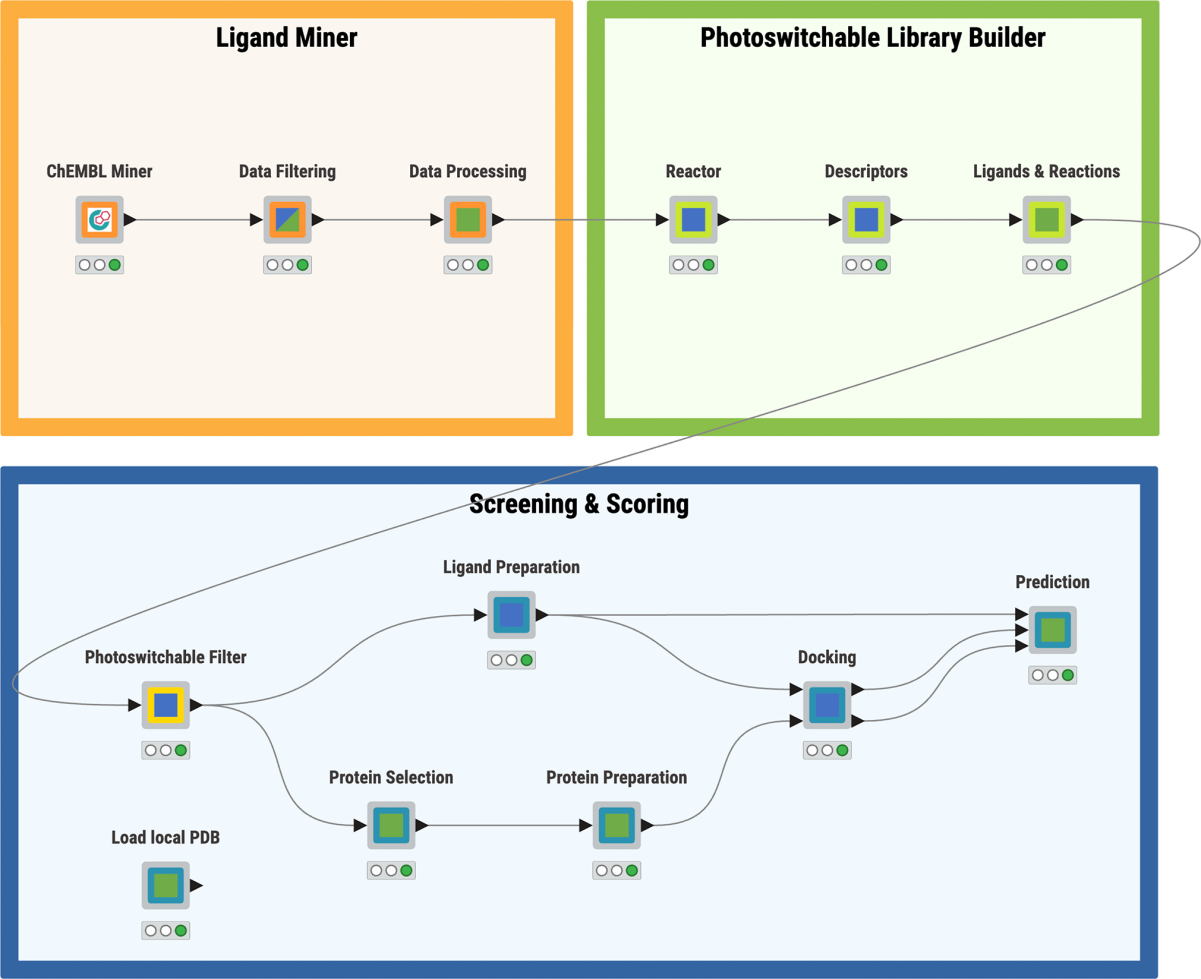
General overview of the PSW-Designer as implemented in the KNIME Analytics Platform. The three main modules are depicted with colored workflow annotations: Ligand Miner (orange), Photoswitchable Library Builder (green), and Screening & Scoring (blue). Component icons indicate an interactive view (green square), configuration window (blue square), or both (blue-green square). A detailed description of each component is available in the Supporting Information.

The PSW-Designer platform comprises three major modules (Figure 2), which can be operated independently: (i) *Ligand Miner*, which retrieves, filters, and processes ligand information and associated binding data from the ChEMBL database,^21^ (ii) *Photoswitchable Library Builder*, which incorporates the azobenzene in an appropriate position of the parent ligand (azoextension) or introduces the azobenzene or azo bond in replacement of suitable functional groups in the parent molecule (azologization), and (iii) *Screening & Scoring*, which is used for virtual screening and scoring of the potential photoswitchable ligands via a structure-based (docking) approach. In this module, the available experimental structures are retrieved from the PDB,^23^ but they can also be complemented by the upload of homology models or AI-generated predictions from AlphaFold.^24^ A detailed description of each module is available in the Supporting Information, while the main aspects are summarized below.

**Figure 3:**
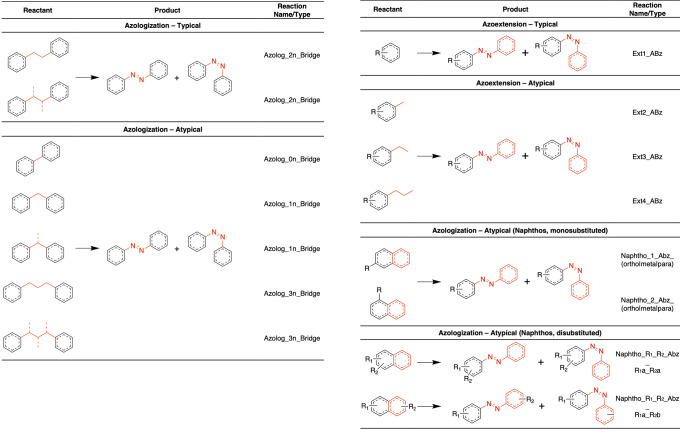
Definitions adopted in the “Reactor” component of the Photoswitchable Library Builder module to define azologization and azoextension SMARTS reactions. The reactant phenyl rings represent any (hetero)aromatic ring systems, with the dotted lines indicating any resonance alternatives, while the single red bonds indicate any aliphatic bond and any atom type within the specification that is replaced by an azo bond.

In this platform, the replacement of a two-atom linker (any atom, any nonbranched linker) connecting two (hetero)aromatic rings by an azo bond is considered a “typical azologization” (Figure 3). Additionally, a series of nonusual or atypical azologization replacements are also included. These correspond to two aromatic rings separated by (i) a single bond, (ii) a one-atom linker, which can have up to one atom-long branching, or (iii) a 3-atom linker, on which each of the atoms can be branched for up to one extra atom. Additionally, a particular case of atypical azologization has also been implemented, in which a mono- or disubstituted naphthalene-like (hetero)aromatic ring is replaced with an azobenzene. For the azoextension reactions—formally, half azoextensions—typical and atypical strategies are employed, with typical azoextension corresponding to the appending of an azobenzene into a free, nonsubstituted position of a (hetero)aromatic ring, and the atypical corresponding to the replacement of a 1- to 3-atoms long substituent by the azobenzene (Figure 3).

## Ligand Miner

The first module (“Ligand Miner”) is composed of three components that retrieve ligand information and associated binding and functional data from the ChEMBL database,^21^ filter the data for quality, process the activity measurements, and classify the data according to user-defined thresholds. Although ChEMBL offers programmatic access via the REST (Representational State Transfer) API service, we opted for incorporating the database connection via a local copy in SQLite format (∼4.2 Gb), which can be downloaded from the ChEMBL website (http://ftp.ebi.ac.uk/pub/databases/chembl/ChEMBLdb/latest/). In the configuration window of the first PSW-Designer component (“ChEMBL Miner”), the user is requested to locate the database.*db* file. This is the only external file necessary for the PSW-Designer execution, and the only moment the user must preconfigure a component (Figure S2a). Once configured, the user can search for a desired protein class, subclass, or family via the component’s interactive view (Figure S2b). In this search and selection window, the user can also filter for desirable organisms or specific protein target(s) within the class, subclass, or family, thus enabling the construction of targeted ligand libraries. After the selection is applied, the component will query the database and retrieve all bioactivity data, assay details, ligand identifiers, and ligand structural information associated with the selected target(s). These data are then passed to the second component, “Data Filtering”, which removes data points outside predefined quality parameters (Table S1), converts the ligand structures from canonical SMILES to the RDKit molecule type, and applies molecular weight and the number of heavy atoms (NHA) filters that can be interactively adjusted. The filtered data are then passed to the third component, “Data Processing”, which retains only the binding affinity measurements and aggregates distinct affinity measurements of each ligand at the specified target into a unified logarithmic measurement “pAffinity” value. In the component’s interactive view, a histogram of the median pAffinity values and a chart of the classified values are displayed, while the user can also interactively filter for target name and organism, specify a desirable range of pAffinity values, and select chemically diverse ligands via hierarchical clustering on Tanimoto fingerprint similarity (Figure S2c).

## Photoswitchable Library Builder

The first component in this module is the “Reactor”, which queries the ligands for predefined scaffolds that are replaced for an azo bond/azobenzene (azologization strategy) or have a half azobenzene appended at a suitable position (azoextension) via SMARTS reactions. The SMARTS transformations and definitions are summarized in Figure 3 and detailed in the Supporting Information, but briefly, azologization reactions are defined by the replacement of a 0–3 atom linker (any atom type, any bond type) separating two aromatic ring systems by an azo bond (N=N), while azoextension entails the appending of a phenyldiazene group (N=N-Ph) to any and all (exhaustive enumeration) nonoccupied position of an aromatic ring or a position occupied by a group of up to one heavy atom (e.g., halogens, hydroxyl, etc.). In the Reactor configuration window, the user can select between azoextension, typical and atypical azologizations, and a special case of atypical azologization on naphthalene-like compounds, which can be either mono- or disubstituted (Figure S3a). In the second component, “Descriptors”, the 2D representations of the designed photoswitchable ligands are aligned to parent compounds for the quick inspection and recognition of the chemical transformation performed by the reactor node. Additionally, relevant physicochemical descriptors, such as logP, polar surface area, molecular weight, fraction sp^3^, etc., are calculated. These descriptors can be selected by the user in the component configuration window. Finally, in the “Ligands & Reactions”, the user can choose between five distinct interactive visualizations of results via the component’s Interactive View panel (Figure S3c, d).

## Screening and Scoring

In the third PSW module, the newly designed photoswitchable ligands are assessed via structure-based virtual screening (docking). Target structural information (when available) is retrieved from the PDB or locally uploaded, proteins, and ligands prepared, and then, ligands are docked and scored using PLANTS.^25^

In the “Ligand Preparation” component, ligands are converted to 3D representation, and the protonation states at physiological pH are generated together with alternative tautomeric states. Subsequently, the geometry is optimized and minimized in the MMFF94^26^ force field using OpenBabel.^27^ For the protein, the user can view a table summary of all available PDB structures in the “Protein Selection” component, together with the PDB IDs, title, resolution, source organism, and a 2D view of all nonprotein molecules bound in each structure (Figure S4a). Alternatively, the user can load a local PDB file—including AlphaFold^28^ predictions—with the “Load local PDB” component. When a selection is made, the PDB structure is loaded into KNIME and displayed. Next, in the “Protein Preparation” component, the user interactively selects the desirable protein chain and the binding pocket ligand, as well as an additional radius for the docking grid sphere (Figure S4b). The component then displays the prepared protein chain and selected binding pocket ligand (Figure S4c). The protein preparation and optimization are done with PDBFixer^29^ and PDB2PQR^30^ Python scripts, which are called from KNIME via the Python Script node.

**Figure 4:**
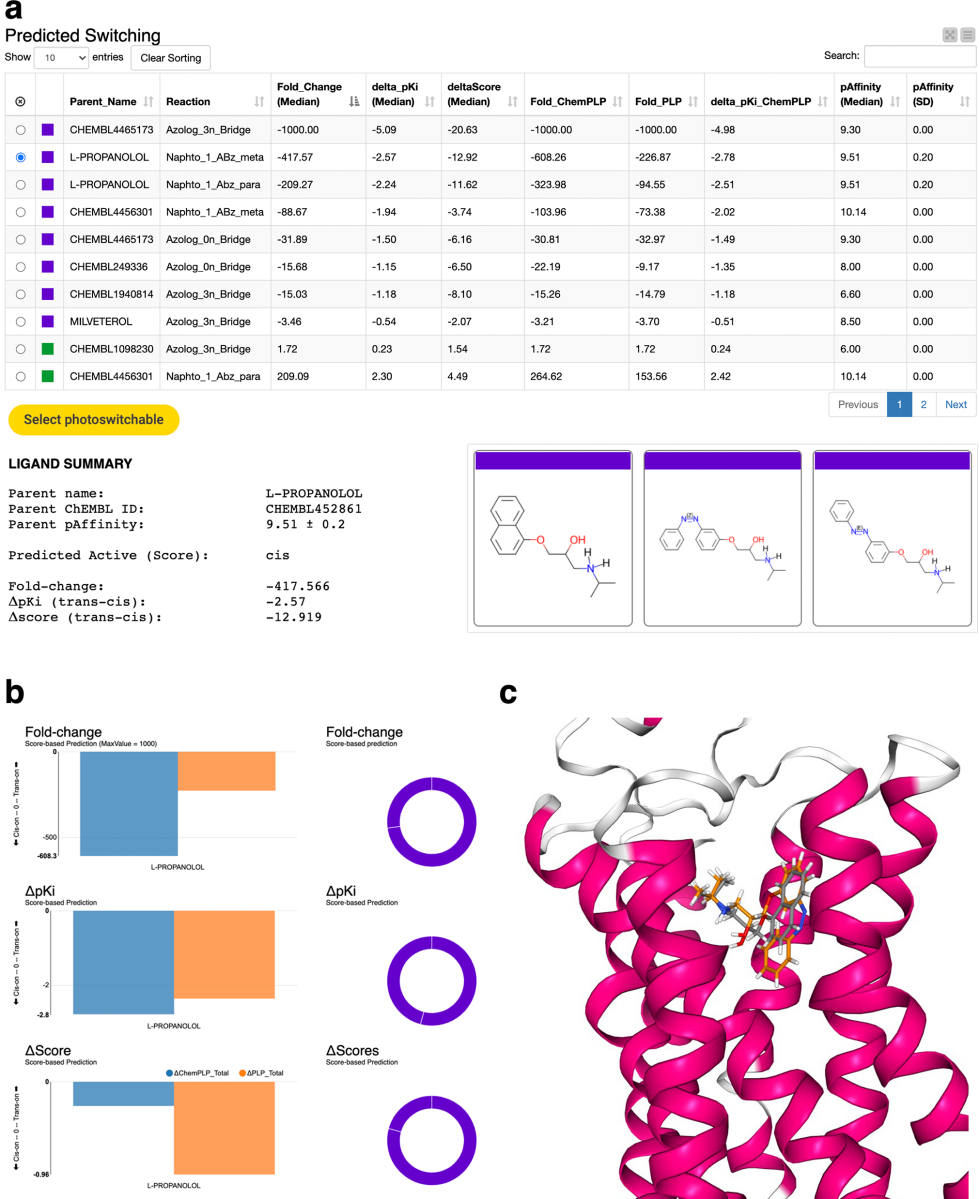
Interactive output of the Screening & Scoring module for photoswitchable ligands for the β_2_-adrenergic receptor. (a) The summary table is sorted by predicted fold-change (descending), with *cis*-ON ligands indicated by negative values and purple coloring and *trans*-ON ligands indicated by positive values and green coloring. (b) Ligand summary, family 2D-depiction, and consensus of fold-change, Δp*K*_i_, and ΔScores for the S-propranolol photoswitchable analog corresponding to Opto-Prop-2, the second highest ranked *cis*-ON ligand in the set. (c) Superimposition of the best-ranked docking binding pose of the parent molecule (S-propranolol, gray) and the *cis*-Opto-Prop-2 (orange) as visualized by the “Prediction” component interactive view.

Once protein and ligands are prepared, and the binding pocket is defined, their respective streams are combined in the “Docking” component. This component runs the PLANTS virtual screening with parallel execution (multiprocessing) with *speed1* and 20 ants, generating 10 poses per ligand variant. In the component’s configuration window, the user can define the number of parallel processes (CPU cores) to be used and the location where the results should be stored. The output table displays the docked poses in SDF format sorted by parent compound name and ChemPLP docking score, together with the docking score energy terms for the ChemPLP and PLP scoring functions.^31^ Finally, in the “Prediction” component, the best pose for each ligand (parent and *trans/cis* photoswitchable ligands) is selected via a Pareto multiobjective optimization. In this process, the poses are ranked by (i) maximizing the number of contacts with the receptor, (ii) minimizing the number of clashes, (iii) minimizing the number of atoms that have no contact with receptor atoms, and (iv) minimizing the ChemPLP total docking score. If more than one Pareto-optimal solution is found, the pose with the lowest score will be retained. Next, for each ligand family (parent molecule, *cis*, and *trans* photoswitchable ligands generated by a specific SMARTS reaction), the differences between the isomer scores (*cis–trans*) are calculated. Then, by leveraging the parent docking score, parent pAffinity measurement, and the difference in score between isomers, a score-based isomeric shift (Δp*K*_i_) and a predicted fold-change (FC = 10^ΔpKi^) are calculated. The median values for ΔScore, Δp*K*_i_, and fold-change are also calculated to define the consensus active isomer (isomer “ON”). In the interactive view, the user receives a table with ligand families, the predicted active isomer, and the estimation of ΔScore, Δp*K*_i_, and fold-change, which can be interactively sorted (Figure 4). A filter for at least 50-fold change between photoisomers is applied as the default, aiming to prioritize ligands that preferentially bind one of its isomeric forms over the other, i.e., maximizing the difference between isomers, as this is often desirable for photopharmacological applications. However, this filter can be interactively changed or completely dismissed by the user.

Once a selection on a ligand family is made, the user is presented with a summary containing the parent ligand name and ChEMBL ID, parent median pAffinity, predicted active isomer, and estimation of isomeric shift, together with a 2D view of the ligand family (Figure 4a). The consensus of the multiple parameters can also be visualized by the interactive bar and doughnut charts (Figure 4b). Finally, the docking poses for the parent and the *trans/cis* photoswitchable pair are displayed in the receptor, allowing for quick inspection of the docking results and visual assessment of prediction (Figure 4c).

## Results

### GPCR Ligand Coverage

In the current release (v32), the ChEMBL database contains approximately 20 million bioactivity measurements for 2.35 million unique molecules, which target more than 15,000 distinct targets. Among these, ChEMBL includes data for 888 class A GPCRs for 21 distinct organisms, of which 367 are human orthologs. For the human class A GPCRs, ChEMBL contains 641,000 data points for 107.356 unique ligands.

The Ligand Miner module in PSW-Designer can retrieve a library with 2.43 M activity measurements for class A GPCRs from the ChEMBL database v32 in 82 s on an iMac computer with a 3.6 GHz Intel Core i9 processor (10 CPU cores) and 32 GB DDR4 memory. After the quality filtering and processing of binding affinity data, a table with 243,602 affinity measurements (pAffinity values) is obtained, comprising 107,383 unique ligands and covering 493 class A receptors from 26 distinct species, of which 198 are human subtypes targeted by 84,771 unique ligands in 184,396 pAffinity measurements.

In the Photoswitchable Library Builder, these 107,383 unique ligands are converted into 26,200 *trans/cis* azologization pairs via classic azologization, 84,542 pairs via atypical azologizations, 221,800 atypical azologizations (naphthalene-like type) and 1.6 million *trans/cis* pairs through azologization reactions (typical and atypical). Moreover, photoswitchable ligands are generated for 85% of the 198 unique class A GPCRs with binding affinity data available on ChEMBL when only typical and atypical azologization reactions are considered, thus virtually covering all of the receptors when naphthalene-like azologizations and azoextension are included.

### Retrospective Case Studies

To evaluate the PSW-Designer performance, particularly for the Screening & Scoring module, we assessed two GPCR targets for which photoswitchable GPCR tool compounds have been published, i.e., β_2_-adrenergic (Opto-Prop-2)^32^ and histamine H_3_ receptors (VUF14738).^33^ Opto-Prop-2 is a *cis*-ON photoswitchable antagonist inspired by the FDA-approved β-blocker Propranolol^34^ (Figure 1b), while VUF14738 is also a *cis*-ON photoswitchable antagonist inspired by CHEMBL476099^35^ (Figure 1c).

### β2-Adrenergic Receptor

Opto-Prop-2 is a photoswitchable tool compound disclosed by Bosma et al. It possesses a 630-fold affinity gain upon *trans-*to-*cis* isomerization, the largest shift reported for a photoswitchable ligand targeting a GPCR (Figure 1b).^32^ To evaluate the design and predictions for Opto-Prop-2 and structurally analogous ligands, we started by collecting all activity data for this receptor subtype with the Ligand Miner. A library of 1747 unique ligands with high-quality affinity measurements for the human β_2_-adrenergic receptor was retrieved and filtered for pAffinity ≥8.0 and chemical diversity (Tanimoto similarity ≤0.50), resulting in a set of 35 parent molecules. These parent compounds were transformed into potential photoswitchable ligands via typical and atypical azologization reactions, including naphthalene-like atypical azologizations (monosubstituted) in the Photoswitchable Library Builder, resulting in a library of 32 *trans/cis* azobenzene pairs. After preparation, the ligands were docked into the XFEL structure of the inactive β_2_-adrenoceptor bound to Alprenolol (PDB ID 6PS2).^36^ This structure was selected for having the highest resolution among the available structures for the β_2_-adrenergic receptor (2.4 Å). The parent and photoswitchable ligands were docked with PLANTS in multiprocessing mode, with the docking grid sphere centered around the experimentally bound ligand with an additional radius of 2.0 Å.

The Prediction component in the Screening & Scoring module suggests 5 of the 16 unique photoswitchable ligands as *trans*-ON and 4 as *cis*-ON when applying a threshold of Δp*K*_i_ > 1.7 or 50-fold for a significant shift between isomers. Among these, Opto-Prop-2 appears as the second highest-ranked *cis*-ON ligand, with a predicted 418-fold isomeric shift in binding affinity (Figure 4a)—the top-ranked has not yet been synthesized, to the best of our knowledge. This value is comparable to the experimentally reported 630-fold (Δp*K*_i_ = 2.8) shift toward the *cis*-isomer. The other two regioisomers reported by Bosma et al. are ranked lower, with the *para*-azo-substituted (relative to the main pharmacophoric group of the parent molecule (*S*)-Propranolol) displaying a 210-fold switch toward the *cis* isomer, while for the *ortho*-substituted analog, a 1000-fold-change (i.e., complete preference) toward the *trans* isomer is predicted. Experimentally, the *ortho-* and *para*-substituted analogs display a 4.3-fold shift toward the *trans* and a 2.6-fold shift toward the *cis* isomer, respectively. Therefore, despite the numeric divergences, the isomeric preference predicted by the PSW-Designer agrees with the experimental results for the other two isomers. Nonetheless, in all cases, (*S*)-Propranolol still ranks high as a potential template ligand, with the platform suggesting a prospective strategy to design new photoswitchable ligands based on this template.

### Histamine H_3_ Receptor

To assess the design and predictions for *cis*-ON histamine H_3_ receptor antagonist VUF14738 (Figure 1c), we retrieved all bioactivity data for this target from ChEMBL. We processed it for binding affinity for the human subtype via the Ligand Miner module. The resulting library of 3717 unique parent compounds was further filtered for high affinity (pAffinity ≥8.0) and chemical diversity (Tanimoto Similarity ≤0.50), resulting in a set of 114 ligands. This parent compound library was then converted into 224 potential *trans/cis* photoswitchable (112 pairs), generated by typical and atypical azologizations—including atypical azologizations for mono- and disubstituted naphthalene-like ring systems. In the Screening & Scoring module, this ligand library was prepared and docked into the recently published X-ray structure of the inactive human histamine H_3_ receptor bound to the antagonist PF-03654746 (PDB ID 7F61).^37^ The docking grid sphere was centered around the experimentally bound ligand and an additional radius of 2.0 Å.

The Prediction component of the Screening & Scoring module indicates that 18 of the 112 unique photoswitchable ligand designs would be *cis*-ON, while 24 would be *trans*-ON, considering a threshold of 50-fold (Δp*K*_i_ ≥ 1.7) for a significant binding affinity shift. For the family of the antagonist ChEMBL476099, which includes 19 distinct photoswitchable ligands due to the exhaustive enumeration of ring substituents, only four ligands are predicted to be *cis*-ON, with isomeric shifts varying between 8- to 490-fold (Figure S5a). Among these is the compound VUF14738, generated from the parent via the ‘Naphtho_2_6_ABz_3a3b’ reaction. VUF14738 is predicted to display an 8-fold change in binding affinity upon *trans*-to-*cis* isomerization (Figure S5b), which is again in agreement with the experimental observation of a modest (13-fold) isomeric shift toward the *cis*-isomer.^33^ The 3D superimposition of the docked poses (parent and photoswitchable ligands) shows that the *cis*-VUF14738 is capable of recapitulating the same anchoring points to the receptor as the parent molecule via the amine and amide groups (Figure S5c), interactions that are disrupted for the *trans*-VUF14738.

## Conclusions

Photopharmacology holds the promise of remotely controlling the action of biologically relevant or even therapeutic targets with light in great spatiotemporal resolution and with minimal disturbances to the biological system and its environment. However, the challenges associated with the multiparameter optimization of often confounding variables such as bioactivity, photochemistry, and physicochemical properties make the design of novel photoswitchable molecules difficult and hinder the potential widespread application of photopharmacology. These challenges are concretely exemplified in the field of GPCR research since; despite being among the most prominent drug targets, GPCRs remain relatively poorly targeted by photoswitchable molecules.^11^

Hence, here we provided a computational platform (the PSW-Designer) that allows the design and structure-based virtual screening of potential new photoswitchable molecules, aiming to aid chemists and (photo)pharmacologists in generating new ligand ideas and hypotheses. This fully open-source workflow, implemented in the KNIME Analytics Platform, takes advantage of bioactivity data for known ligands deposited in ChEMBL, curates these data, and applies molecular transformations to these template molecules following strategies that have been experimentally successful in generating photoswitchable molecules. These libraries can then be virtually screened to prioritize ligands for synthesis, allowing the estimation of isomeric shifts and the prediction of the active isomer by exploiting parent ligand bioactivity information.

We demonstrate the PSW-Designer applicability by generating a library of potential photoswitchable ligands that covers two-thirds of all druggable class A GPCRs (198 receptors), in contrast to the ∼30 receptors currently targeted experimentally by photoswitchable molecules. Moreover, we assessed the predictive performance of the PSW-Designer using two retrospective studies on photoswitchable antagonists for the β_2_-adrenergic and histamine H_3_ receptors. In both test cases, the PSW-Designer was able to predict the active isomer and estimate the affinity isomeric switches (Δp*K*_i_) in accordance with experimental results. While the retrospective study identified compounds for which we have already described syntheses,^32,33^ for prospective studies, the selection of top-ranking compounds will have to be followed by an inspection of synthetic feasibility. Thorough retrosynthetic analyses will further refine the priority of the top-ranking compounds, as the insertion of a photoswitchable moiety in a template ligand delivers a new chemical entity and often leads to significant changes in the synthetic route used for the template ligand.^11^

While we chose to focus on class A GPCRs here for demonstration and validation purposes, the PSW-Designer can be applied to any protein target with known ligands and bioactivity data and with experimental or modeled structure. Thus, we anticipate that this platform can assist the design of novel photoswitchable molecules for various targets including ion channels, transporter proteins, or enzymes such as kinases and hydrolases. Although we focused on the azobenzene moiety due to its wide application in photopharmacology, robust photochemistry, and straightforward synthetic accessibility, support for other photoisomerizable groups will be implemented in a future version of the PSW-Designer.

Also, by providing open-source alternatives to routine cheminformatics and computational drug design tasks to be performed from within KNIME, we contribute to the KNIME computer-aided drug design (CADD) community by filling a void of tools for critical steps in any CADD campaign, such as ligand and protein preparation and multiprocessor high-throughput docking. Finally, this platform will help guide the design of photoswitchable ligands for GPCRs and other protein targets, with improved photopharmacological properties and increased isomeric binding affinity shifts—thus helping to establish photopharmacology as a viable tool for the modulation of biological targets and the survey of physiology in real-time, both *in vivo* and *in vitro*, and eventually into the clinics.

## Data Availability

PSW-Designer is available free of charge via the Knime Hub (https://hub.knime.com/icarosimon/spaces/PSW-Designer/latest/) and is available for Linux and OS platforms. PSW-Designer has been extensively tested in MacOS Ventura 13.4 and RockyLinux 9.2 operational systems with KNIME Analytics Platform v 4.7.4.

## Supporting Information Available

Detailed description of each module and component of PSW-Designer. Supplementary figures and tables: output of PSW-Designer when applied to the histamine H_3_ receptor (PDF)
