# Supplementary material for: PSW-Designer: An Open-Source Computational Platform for the Design and Virtual Screening of Photopharmacological Ligands
Source: J Chem Inf Model. 2023 Oct 13;63(21):6696–705. doi: 10.1021/acs.jcim.3c01050 (PMC10647043; doi:10.1021/acs.jcim.3c01050)
Supplement: Supplementary file 1 — ci3c01050_si_001.pdf [file ci3c01050_si_001.pdf]

PSW-Designer: An Open-Source Computational Platform for the Design and Virtual Screening of Photopharmacological Ligands

SUPPORTING INFORMATION

Icaro A. Simon[†], *Evert J. Homan*[‡], *Maikel Wijtmans*[†], *Michael Sundström*[§], *Rob Leur*[†], *Iwan J.P. de Esch*[†], *Barbara A. Zarzycka*[†]

[†]*Division of Medicinal Chemistry, Faculty of Science, Amsterdam Institute for Molecular and Life Sciences, Vrije Universiteit Amsterdam, 1081 HZ Amsterdam, The Netherlands.*

[‡]*Science for Life Laboratory, Department of Oncology-Pathology, Karolinska Institutet, S-171 76 Stockholm, Sweden*

[§]*Centre for Molecular Medicine, Karolinska Institutet, S-171 76 Stockholm, Sweden*

Detailed Platform Description

The PSW-Designer is implemented in the KNIME Analytics Platform (v. 4.7.3) and is composed of three independent modules and 13 components (Figure S1). With small adjustments, these modules can be decoupled to perform their tasks on alternative datasets and/or distinct KNIME workflows. The configuration details of each module and its components are detailed below.

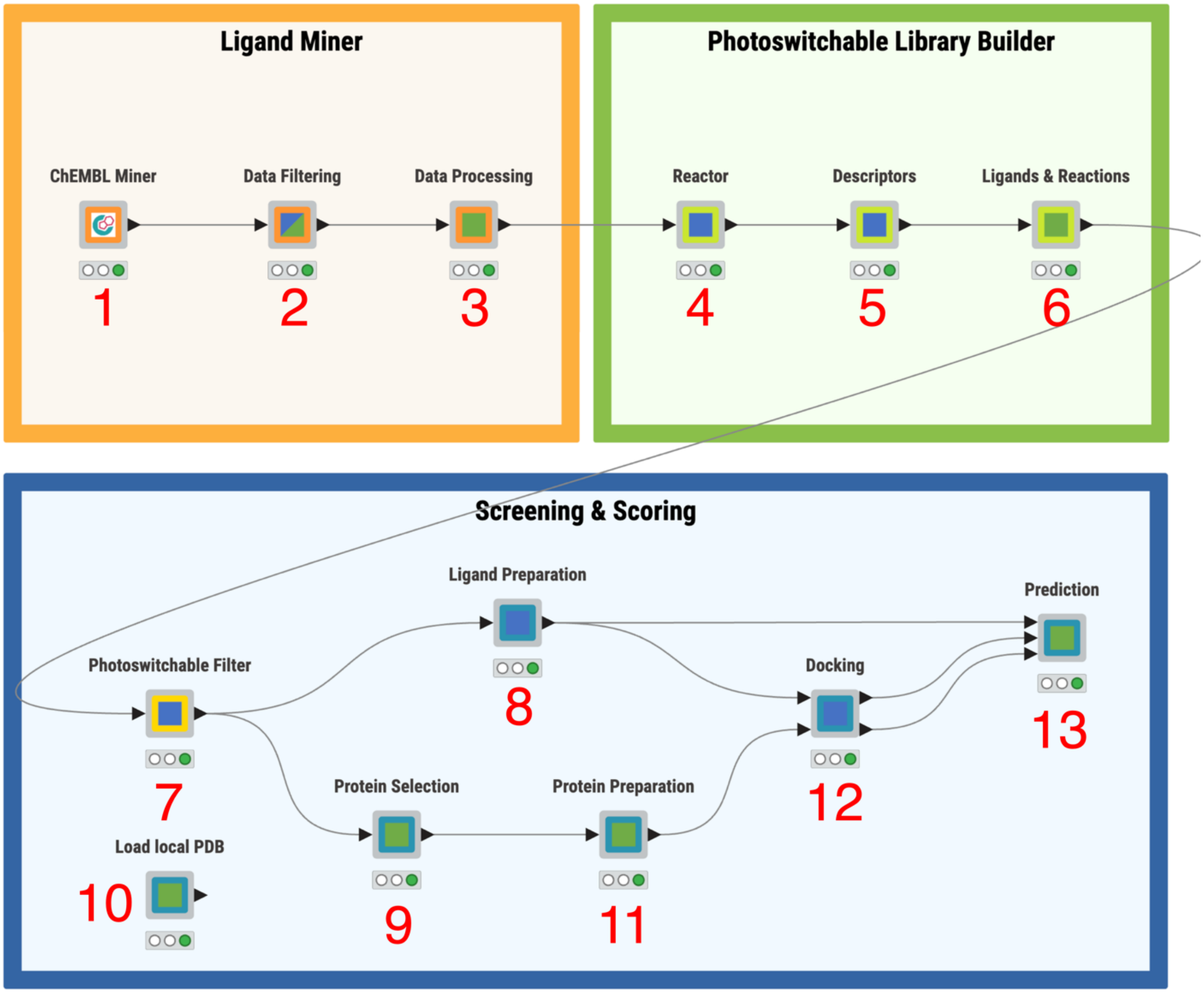

Figure S1. General overview of the PSW-Designer as implemented in KNIME Analytics Platform. The "Ligand Miner" module is composed of three components that (1) retrieve ligand and activity information from the ChEMBL database, (2) filter this data for quality, and (3) process this data for binding affinity. The "Photoswitchable Library Builder" is also composed of three component that (4) performs the azologization and azoextension reactions, (5) calculate physicochemical descriptors, and (6) displays the reaction results. Finally, in the "Screening & Scoring" module, which has seven components, (7) the ligands are filtered and (8) prepared, (9) protein coordinates are retrieved, and (11) receptors are prepared for (12) a structure-based (docking) screening of the newly generated photoswitchable ligands and (13) the estimation of isomeric shifts and binding affinity.

Ligand Miner

This first PSW-Designer component, **ChEMBL Miner** (Figure S1-1), establishes a connection to a local copy of the ChEMBL[1] database via an SQLite Connector node. The location of the database can be adjusted at the component configuration window. Next, the protein classification information is retrieved from the database (eight classification levels) and concatenated (unpivoted) into a searchable column, allowing the user to interactively retrieve all ChEMBL data for a protein class, family, or subfamily of interest in the component's interactive view. Based on the user selection, the database is queried on the desired protein classification level. All activity measurements, assay details, target information, ligand identifiers, and ligand structural information are retrieved. The ligand information is collected for parent compounds, i.e., the molecules stripped of salts, via the "*parent_molregno*" database field. Also, via nested "Interactive Value Filter Widget", the user can interactively select organisms/species, and specific targets through the components view. If available, the molecule will be renamed to its common name, else the "ChEMBL ID" will be used as the identifier.

Table S1. The quality-based filter implemented in the "Data Filtering" component. A "Rule-based Row Filter" excludes TRUE matches to the following conditions.

MISSING $canonical_smiles$ => TRUE
$confidence_score$ < 6 => TRUE
$activity_comment$ = "Not Determined" => TRUE
$activity_comment$ = "Not Evaluated" => TRUE
$activity_comment$ = "No data" => TRUE
$activity_comment$ = "Nd(Toxic)" => TRUE
$activity_comment$ = "Nd(Insoluble)" => TRUE
$activity_comment$ = "ND(Insoluble)" => TRUE
$activity_comment$ = "NT" => TRUE
$activity_comment$ = "NC" => TRUE
$activity_comment$ = "Insoluble" => TRUE
NOT $potential_duplicate$ = 0 => TRUE
NOT MISSING $data_validity_comment$ => TRUE
$activity_comment$ = "Inconclusive" AND (MISSING $standard_value$ OR MISSING $pchembl_value$) => TRUE
$activity_comment$ = "inconclusive" AND (MISSING $standard_value$ OR MISSING $pchembl_value$) => TRUE

The second component, **"Data Filtering"** (Figure S1-2), removes data points that have (*i*) ChEMBL confidence score <6.0, (*ii*) missing *SMILES* structure information, (*iii*) are assigned as potential duplicates, or (*iv*) are assigned as "not evaluated" or "not determinate", etc., in the activity comment (Table S1). This component also converts canonical *SMILES* obtained from ChEMBL into *RDKit*[2] molecule type, with partial sanitization to perceive aromaticity and correct stereochemistry. It also calculates the molecular weight and number of heavy atoms (NHA) via the RDKit Descriptor Calculate node. These two descriptors are used in the component's interactive view, in which a filter for NHA ≤ 40 is applied by default, as larger molecules slow downstream processing significantly. However, the user can adjust this threshold or dismiss the filter in the interactive view (Figure S2-c), where histograms for these two descriptors are also displayed. In the component configuration dialog, the user can select which data columns (i.e., ChEMBL database fields) to keep in the KNIME data table during the following steps of workflow execution.

Table S2. Filter for affinity measurements implemented in the "Data Filtering" component. A "Rule-based Row Filter" includes only TRUE matches to the following conditions.

```
($assay_type$ = "B") AND ($standard_type$ IN ("Ki", "pKi", "Kb", "pKb", "Kd", "pKd", "KB", "pKB", "IC50", "pIC50", "logIC50", "logKi")) => TRUE
($assay_type$ = "B") AND ($standard_units$ = "nM") AND ($standard_type$ IN ("Affinity", "Binding affinity", "Activity")) => TRUE
```

In the third component of the Ligand Miner module, "**Data Processing"** (Figure S1-3), the binding affinity data is filtered from functional data if "standard type" and "standard units" are signed according to a predefined rule (Table S2). Data on mutant targets are also excluded. Next, different affinity measurements (e.g., K_i, K_b, K_d, IC_{50}, pK_i, pIC_{50}, etc.) for the same ligands (via Ligand ChEMBL ID) at the same target (via Target ChEMBL ID) are joined into a unified logarithmic measurement "pAffinity" (Table S3). For ligands for which only IC_{50} is available, the approximation that $K_i = IC_{50}/2$ is applied. This approximation is taken from the Cheng-Prusoff equation with the assumption that the applied concentration of the labeled ligand is similar to its K_d.[3] After removing potential missing and infinite values, the median, standard deviation, minimum and maximum values, and the total count of measurements per ligand and per target are calculated. This approach allows for a more accurate estimation of ligand binding and a larger ligand coverage than if pChEMBL values were used. Finally, the ligands are classified into "actives" (binders), inactive (nonbinders), and "intermediate" using user-predefined thresholds assigned via the component configuration. In the component's interactive view, a histogram of the median pAffinity and a chart of the classified values is displayed, together with interactive filters for species and target – if not preselected in the first component of the Ligand Miner node (Figure S2-c).

Table S3. Parameters and approximations to calculate the pAffinity value. These definitions are implemented in the "Data Processing" component, via a Column Expressions node.

```
if (not(isMissing(column("standard_value")))) {a = abs(column("standard_value"))}
if ((column("standard_units") == "nM") && regexMatcher(column("standard_type"),("Ki|Kd|KB|Kb|Activity|Binding affinity"))) {-1*log(a/1E9)};
if ((column("standard_units") == "nM") && (column("standard_type") == "IC50")) {-1*log(a/2E9)};
if (column("standard_type") == "logIC50") {a+log(2)};
if (regexMatcher(column("standard_type"),"pKi|pKb|pKB|logKi")) {a};
if (not(isMissing(column("pchembl_value")))) {b = column("pchembl_value")}
if (column("standard_value")== 0 && not(isMissing(column("pchembl_value"))) &&
regexMatcher(column("standard_type"),("Ki|Kd|KB|Kb|pKi|pKb|pKB|logKi"))) {b};
if (column("standard_value")== 0 && not(isMissing(column("pchembl_value"))) && regexMatcher(column("standard_type"),("IC50|logIC50")))
{b+log(2)};
if (not(isMissing(column("activity_comment"))) && regexMatcher(column("activity_comment"),"^Not.+")) {5};
```

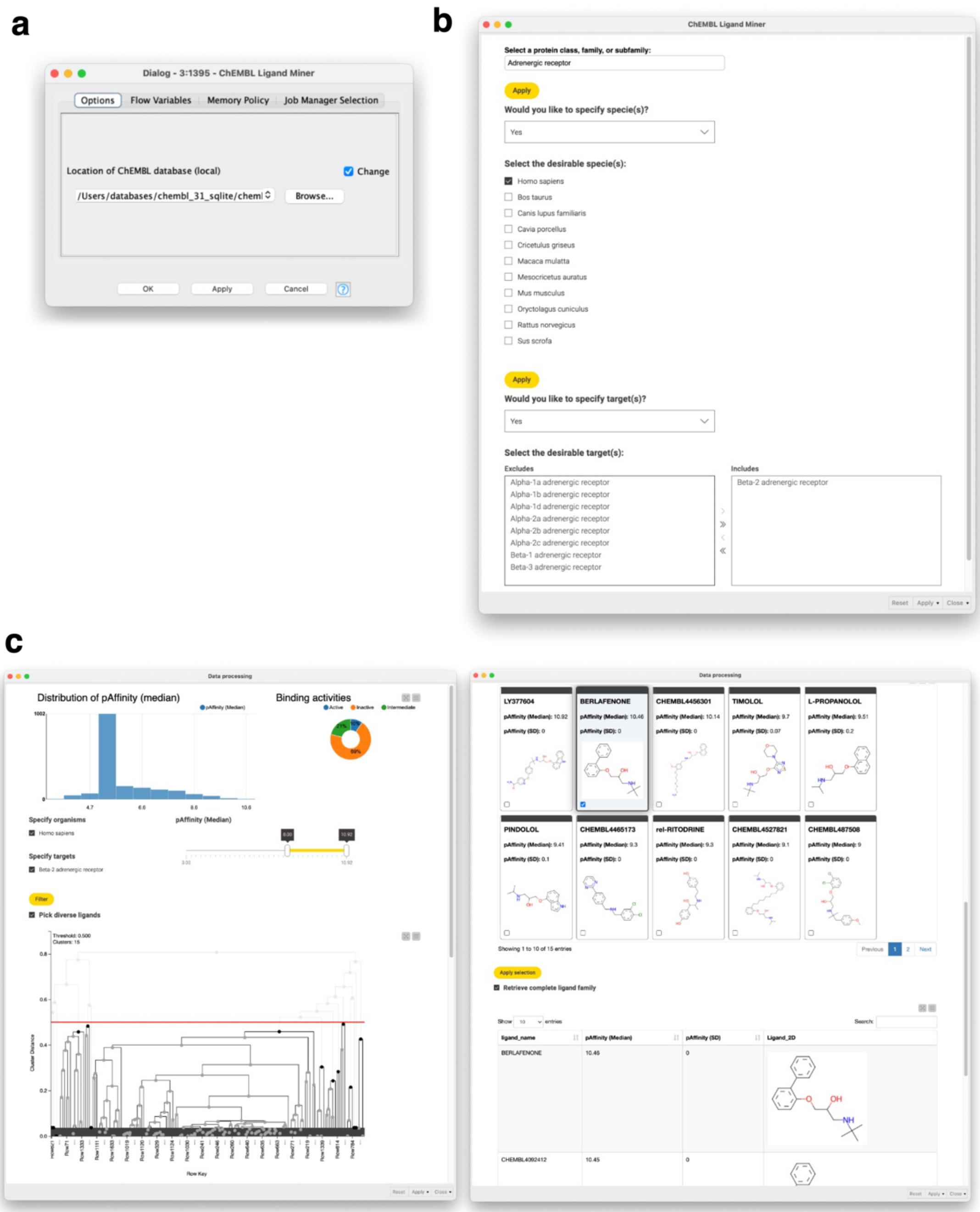

Figure S2. Configuration windows and interactive views of the Ligand Miner module. a. Configuration window of the ChEMBL Miner (top), where the user can browse and connect to a local copy of the ChEMBL database in SQLite format. This is the only pre-configuration required for PSW-Designer execution. **b.** The interactive view of the ChEMBL Ligand Miner component, where the user can interactively search for protein class, family, and subfamily. Once the selection is applied, the data is retrieved, and the user can filter for specific organisms or target subtype(s). **c.** Interactive view of the "Data Processing" component displaying a histogram of pAffinity values and a chart of the number of ligands classified as active, inactive, and intermediate according to user-defined thresholds, specified in the component's configuration window. In this interactive view, the user can also interactively filter for pAffinity ± SD and choose to retrieve a chemically diverse set of ligands defined via a threshold of Tanimoto fingerprint similarity assigned through the hierarchical clustering view (left panel). The highest affinity ligand in each cluster is depicted in an interactive grid (right panel). Alternatively, the user can select ligands in this grid and retrieve all other ligands in the cluster, i.e., all similar ligands (right panel).

Photoswitchable Library Builder

In this module, the "**Reactor**" component (Figure S1-4) starts by grouping the parent ligands by their ChEMBL ID to remove potential duplicates and then filtering the molecules for the appropriate template query, which varies for the five distinct reactions employed. This *SMARTS* substructure filter is applied to also remove any already existing photoswitchable molecules from the template list, else the parent molecule and one of the isomers would be the same chemical entity. Next, the "RDKit One Component Reaction" node is used to perform the *SMARTS* transformations, which are looped over all ligands and all transformations for the specified reaction type. In the *SMARTS* transformation, both isomers *trans/cis* (E/Z) are generated with the configuration appended into an "Isomer" column and the reaction type/name appended to a "Reaction Type" column. Canonical *SMILES* are generated for the product molecules and used for duplicate filtering, hence removing potential duplicate products due to molecular symmetry, while RDKit-type fingerprints (2048 bits) are generated for both parent molecules and products. Likewise, the "RDKit Descriptor Calculation" node is applied to calculate the number of heavy atoms (NHA). Finally, the results are filtered according to (*i*) Tanimoto fingerprint similarity (azologization reactions), (*ii*) max allowed variation in the NHA, i.e., NHA_{parent}-$NHA_{product}$ (azologizations and azoextensions) to prevent products that may lack critical functional groups. Although the user can alter these filter thresholds via the configuration window of the subcomponents, we recommend the default values of ≥ 0.2 and ≤ 3.0 for the Tanimoto fingerprint similarity and change in NHA, respectively. For the naphthalene-like azologization, a change in molecular weight is used as a filter instead, excluding products with a reduction in molecular weight.

***SMARTS* Definitions.** In the PSW-Designer platform, a two-atom-linker connecting two (hetero)aromatic rings is considered a prototypical azoster (i.e., a typical azologization, with reaction name "Azolog_2n_Bridge", Figure 3). Such definition is considered a prototypical structural feature of azoster, and this strategy has been extensively employed in the design of photoswitchable ligands.[4,5] More recently, Koubari *et al.* (2021) have shown that diaryl sulfonamides tend to adopt a conformation in a solution that resembles *cis* azobenzene, and can therefore be considered "cisoid azosters".[6] To include this and related functional groups in the *SMARTS* reactions, we also allowed for a one atom-long branch on the linker atoms. Additionally, a series of non-usual or atypical azologs is also included in the queries. These correspond to two aromatic rings separated by (*i*) a single bond (reaction name: Azolog_0n_Bridge), (*ii*) a one-atom linker, which can have up to one atom-long branch (reaction name: Azolog_1n_Bridge), or (*iii*) a 3-atom linker, on which each of the atoms can be branched for up to one extra atom (reaction name: Azolog_3n_Bridge).

For the "naphthalene-like" atypical azologization, two subcases are considered, of mono- and disubstituted naphthalene rings. Due to molecular symmetry, naphthalene-like mono-substitution can only occur in positions 1 and 2 of the ring system. These positions are mapped to the *ortho*, *meta*, or *para* positions of the azobenzene product, with all three substitution patterns enumerated during product generation. For the disubstituted ring systems, the position of substituents in the template ligand is mapped to compose the reaction name. The substituents are appended to the same ring ($R_1a\_R_2a$) or

distinct rings (R_1a_R_2b), with substituent enumeration referenced to the azo bond. All possible combinations of the substituents' positioning around the azobenzene rings are enumerated. For the azoextension reactions – formally, half-azoextensions – typical and atypical strategies are employed simultaneously, with *typical azoextension* corresponding to the appending of an azobenzene into a free, non-substituted position of a (hetero)aromatic ring (reaction name: Ext1_ABz) and the *atypical azoextension* corresponding to the replacement of a 1- to 3-atoms long substituent (reaction name: Ext[2-4] ABz) by the azobenzene. All *SMARTS* queries include any atom and any bond type, with the sole requirement that the linker bridges two aromatic ring systems.

In the second component of the Photoswitchable Library Builder module, **"Descriptors"** (Figure S1-5), the 2D representations of photoswitchable ligands are aligned to their respective parent compounds, which allows for the quick inspection and recognition of the chemical transformation performed by the reactor node. The aligned molecules are then rendered to *SVG* via the "Renderer to Image" node for further use. Additionally, the "RDKit Descriptor Calculator" node is applied to calculate relevant physicochemical descriptors, such as *S*logP, TPSA, molecular weight, fraction SP_3, etc. In the component configuration window, the user can select among 28 distinct descriptors that can be calculated.

Finally, in the last component of this module, "**Ligands & Reactions"** (Figure S1-6), the user can choose between five distinct interactive visualizations of results via the component's Interactive View panel (Figure S3). Those visualizations include (*i*) *Reactions*, in which the user can filter for and inspect the chemical transformations performed in the template compound, together with relevant information for the parent compound, such as ligand name, biological target, and pAffinity values; (*ii*) *Azologs* and (*iii*) *Photoswitchables*, in which the user can visualize a list of template molecules and products, respectively, and interactively sort the list by experimental values and/or calculate physicochemical properties; and (*iv*) *Azologs by Target*, and (*v*) *Photoswitchables by Target*, which allow the inspection of template and product molecules, respectively, but with the additional possibility of filtering for a protein target and organism – if this selection has not been made previously in the Ligand Miner module – and also allowing for the interactive plotting of any previously calculated descriptors or the experimental affinity, enabling the selection of photoswitchable product molecules that satisfy user-specified thresholds of desirable physicochemical properties to be taken to the Screening & Scoring module.

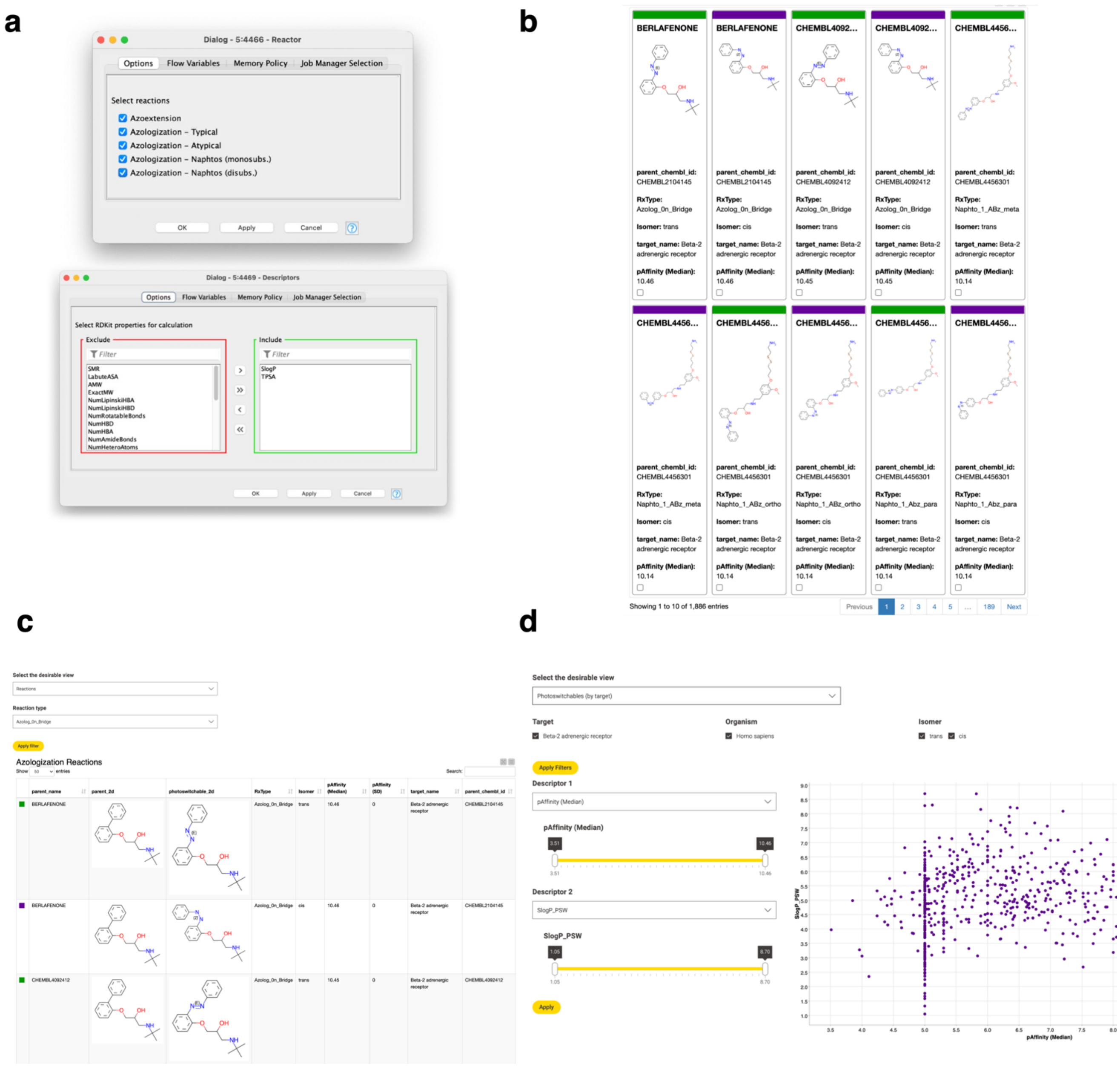

Figure S3. Configuration windows and interactive views of the Photoswitchable Library Builder module. a. Configuration window of the "Reactor" component (top) displaying the typical and atypical azologization and azoextension SMARTS reactions available, and configuration window of the "Descriptors" component (bottom), where the user can select among 28 distinct physicochemical descriptors to be calculated for parent and photoswitchable ligands. **b-d.** Selected interactive views from the "Ligand & Reactions" component, displaying (**b**) a grid of photoswitchable ligands, (**c**) the reactions, and (**d**) the interactive scatter plot of selected physicochemical properties/descriptor and/or experimental measurements (for parent compounds).

Screening & Scoring

In the first component of this module, "**Photoswitchable Filter**" (Figure S1-7), the user has one extra opportunity to interactively filter the newly generated ligands according to the target protein, species/organisms, pAffinity threshold and/or reaction type. Also, via a series of nested "Interactive Value Filter" widgets, the user can add up to five distinct filters for any numeric property, either experimental (for parent molecule) or calculated – i.e., physicochemical descriptors for parent or photoswitchable ligands.

Next, the ligands are prepared for docking via the "**Ligand Preparation"** component (Figure S1-8). In this component, parents and photoswitchable ligands are converted from the 2D RDKit molecular representation to 3D (in *SDF* format) via the "MolConverter" node (ChemAxon Ltd). In this step, the hydrogens are added, and the molecular geometry is quickly optimized in the MMFF94 force field[7]. Next, the protonation states at physiological pH and tautomers are generated via the "Tautomer Generator (Naomi)" node (BioSolveIT GmbH).[10]/11/23 2:55:00 PM The number of distinct microstates per ligand carried out for docking can be adjusted at the component's configuration, with the default value of one protomer/tautomer per ligand selected via a ranking on formal charge requirements. If multiple protomers and tautomeric states are allowed, the ligands are renamed accordingly. Finally, the ligand geometry is optimized via a conjugate gradient energy minimization in the MMFF94 force field via OpenBabel[8], which is also used to convert the ligands to *mol2* type required for the next step, docking in PLANTS.[9]

In the protein target stream, the first component is "**Protein Selection"** (Figure S1-9) sends API request to Uniprot[10] via the target Uniprot ID, querying information on gene name, organism, protein names, and cross-referencing to the Protein Data Bank (PDB)[11,12]. In the component's configuration window, the user specifies if the query should be performed via Uniprot accession number (i.e., for a single organism) or by gene name, i.e., retrieving all structures available from all organisms – the default option. The Uniprot API response is processed into a list of PDB IDs associated with the requested target(s), from which the PDB structural information, heteroatom information, and general properties are retrieved using the "RCSB PDB Tools" node from Vernalis (Vernalis Research Ltd.).[13] Lastly, additional data processing is performed to display the information in the interactive view, while the selected protein is displayed with the "Proteins Viewer" node of 3D-e-Chem.[14] Alternatively, if the target experimental structure is not available, an AI-generated structure prediction can be downloaded from AlphaFold[15] and loaded locally into KNIME via the **"Load Local PDB"** component, or a homology model can be imported via the "**Load Local PDB"** component (Figure S1-10). In this component's interactive view, the user locates a PDB file within their filesystem, which is loaded through the "Load Local PDB Files" Vernalis node and displayed via the 3D-e-Chem's "Proteins Viewer" for visual inspection.

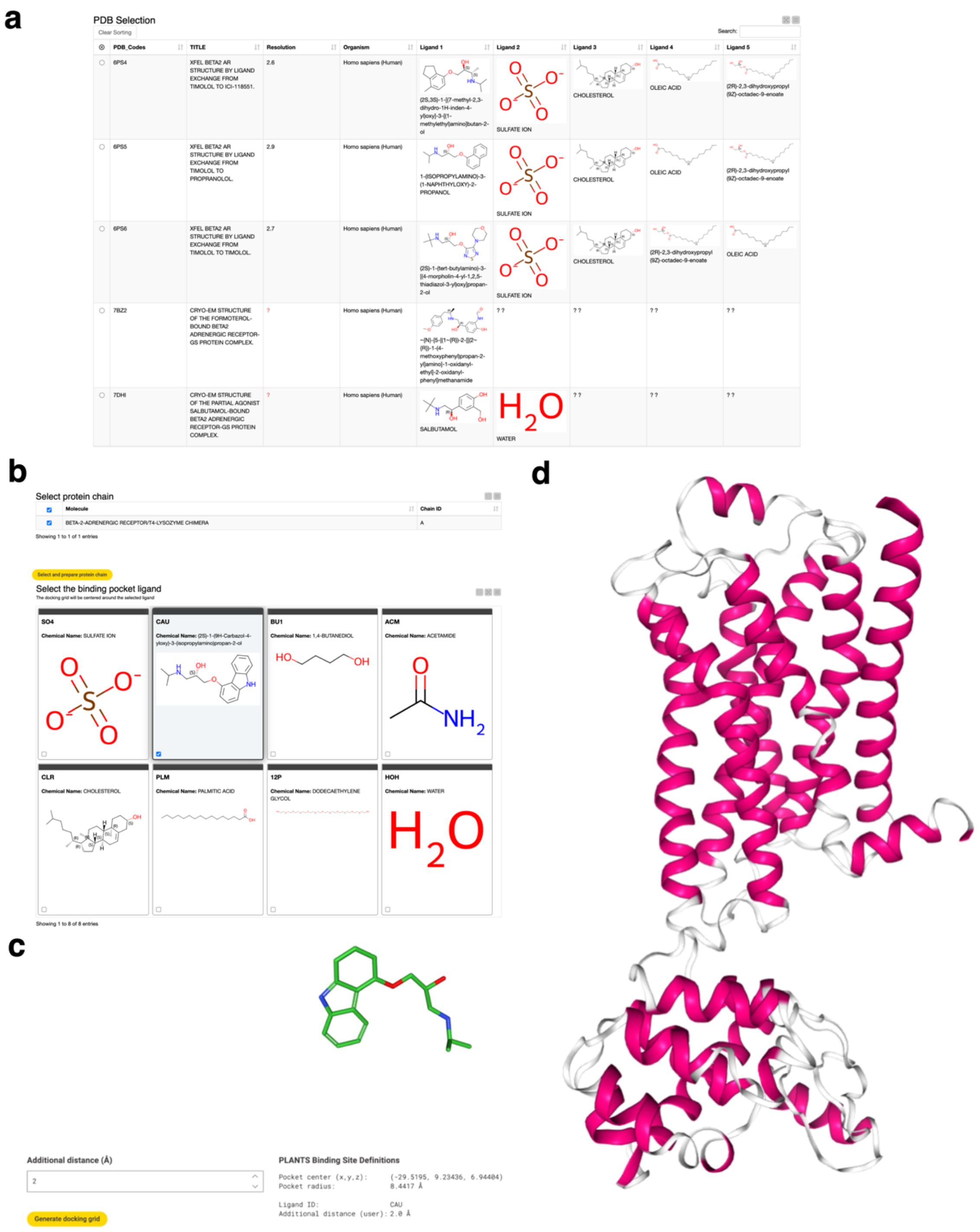

Figure S4. Interactive views of the "Protein Selector" and "Protein Preparation" components. a. Interactive view of the "Protein Selector" component displaying all PDB structures available for a desirable protein target with PDB ID, title, resolution, source organism, and the chemical structure of all bound ligands molecules (heteroatoms). **b-d.** Interactive view of the "Protein Preparation" component. Once a selection is made in the "Protein Selector", the user is presented with an interactive table to select the desired protein chain (**b**, top), and then selects the binding pocket ligand (**b**, bottom). The selected ligand is immediately displayed (**c**) together with the PLANTS docking grid information, which can be adjusted by the inclusion of additional distance. (**d**) The 3D structure of the prepared protein is displayed in an interactive view for quick inspection (side chains omitted for clarity).

The second component in the protein stream, “**Protein Preparation**” (Figure S1-11), retrieves and displays information about the protein chains in the selected PDB structure. In the case of ligand(s)-protein complexes, it also splits the coordinates of ligands from the protein coordinates and extracts the selected protein chain using the “Regex Extractor” node from the “Palladian for KNIME” text processing tools. By the PDB identifiers, the chemical structures and information of all ligand molecules (heteroatoms) present in the PDB structure are retrieved via the Vernalis “PDB Describe Heterogens” node and displayed at the interactive view for user selection of the ligand binding pocket. Once the ligand binding pocket is selected, its 3D coordinates are extracted from the PDB and converted to *mol2* via OpenBabel and used to define the centroid of a sphere that circumscribes the docking grid for PLANTS[9,16] Runner node. At this stage, an additional radius can be added via the interactive view to expand the docking grid and allow docking of larger molecules than the crystallographic/experimental ligand. Meanwhile, the selected protein chain is prepared and optimized using PDBFixer[17] and PDB2PQR[18] Python scripts, which are called into KNIME via the Python Script node. With PDBFixer, the heavy atoms of missing side chains are added, non-standard residues are converted to standard types, water and all nonprotein atoms are removed, and the missing hydrogens are added at physiological pH. Next, PDB2PQR is used to assess the protonation states at pH 7.4 with ProPKA[19], to relax atoms in close proximity in the CHARMM[20] force field, and to optimize the hydrogen bond network. Finally, OpenBabel is used to convert the output *PDB* to *mol2*, a requirement for the PLANTS docking engine.

Once receptor and ligands are prepared and the binding pocket is defined, their respective streams are combined in the “**Docking**” component (Figure S1-12). This component runs the PLANTS[16] virtual screening with parallel execution (multiprocessing), with *speed1* and 20 ants, generating 10 poses per ligand variant. In the component’s configuration window, the user can define the number of parallel processes (CPU cores) to be used and the location where the results should be stored. The output table displays the docked poses in SDF format – after conversion from *mol2* via ChemAxon’s “MolConverter” node – and sorted by parent name and ChemPLP docking score, together with the docking score energy terms for the ChemPLP and PLP scoring functions.[16]

In the final component, “**Prediction**” (Figure S1-13), the best pose for each ligand (parent and *trans/cis* photoswitchable ligands, including alternative protonation and tautomeric states) is selected via a Pareto multiobjective optimization. In this process, by looping over the ligand names, the poses are ranked by (*i*) maximizing the number of contacts with the receptor, (*ii*) minimizing the number of clashes, (*iii*) minimizing the number of atoms without contacts with receptor atoms, and (*iv*) minimizing the ChemPLP total docking score. If more than one Pareto optimal solution is found, the lowest score one will be retained. Next, each ligand family – parent molecule plus the *trans/cis* photoswitchable ligands generated by each specific *SMARTS* reaction – are appended to the same table row, and the differences between the ChemPLP and PLP docking scores (i.e., difference *cis*–*trans*) are calculated. Then, assuming the docking scores as a surrogate to the binding free energy (ΔG), and therefore a linear relationship between the docking score and the natural logarithm of a ligand’s binding affinity, the isomeric shift (ΔpK_i) can be estimated according to:

$$\Delta pK_{i_{(cis-trans)}} = \frac{Score_{cis} - Score_{trans}}{Score_{parent}} * pAffinity_{parent}$$

Where *Score* represents the total docking score of the ChemPLP and PLP scoring functions, independently used to estimate the isomer shift. These isomeric shifts are then used to calculate the predicted fold-change (FC = $10^{\Delta pKi}$), which is limited to the boundaries of -1000 (*cis*-ON only) and +1000 (*trans*-ON only). The median values for ΔScore, ΔpK_i, and fold-change are also calculated and used to define the consensus active isomer (isomer "ON"). The additional nodes process the calculated data and the selected docking poses to be displayed in the interactive view (Figure 4). In this view, the user receives a table with ligand families, the predicted active isomer, and the estimation of ΔScore, ΔpK_i, fold-change, and other calculated predictions, which can be interactively sorted. Once a selection for a ligand family is made, the user is presented a summary with parent ligand name and ChEMBL ID, median pAffinity and standard deviation, the predicted active isomer and estimation of isomeric shift, together with a 2D view of the ligand family (Figure S5a,b). The consensus of the multiple parameters can also be visualized by the interactive bar and donut charts. Finally, the docking poses for parent and *trans/cis* photoswitchable pairs are displayed within the receptor, allowing for the visual inspection of docking results and assessment of predictions (Figure S5c).

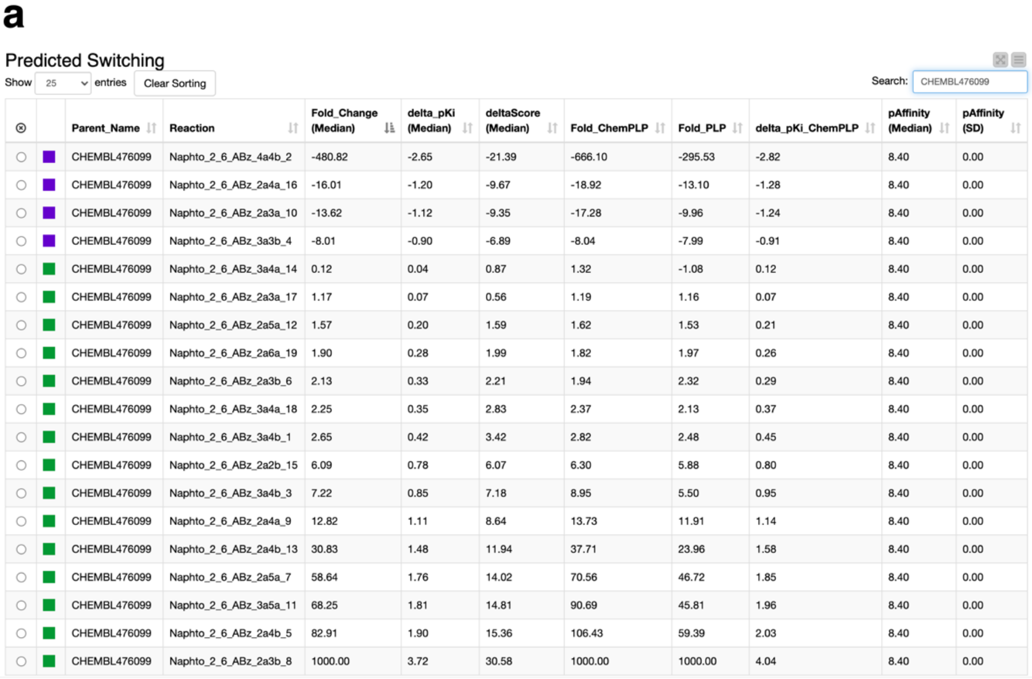

a

Predicted Switching

Show 25 entries Clear Sorting Search: CHEMBL476099

⊗		Parent_Name	Reaction	Fold_Change (Median)	delta_pKi (Median)	deltaScore (Median)	Fold_ChemPLP	Fold_PLP	delta_pKi_ChemPLP	pAffinity (Median)	pAffinity (SD)
○	■	CHEMBL476099	Naphto_2_6_ABz_4a4b_2	-480.82	-2.65	-21.39	-666.10	-295.53	-2.82	8.40	0.00
○	■	CHEMBL476099	Naphto_2_6_ABz_2a4a_16	-16.01	-1.20	-9.67	-18.92	-13.10	-1.28	8.40	0.00
○	■	CHEMBL476099	Naphto_2_6_ABz_2a3a_10	-13.62	-1.12	-9.35	-17.28	-9.96	-1.24	8.40	0.00
○	■	CHEMBL476099	Naphto_2_6_ABz_3a3b_4	-8.01	-0.90	-6.89	-8.04	-7.99	-0.91	8.40	0.00
○	■	CHEMBL476099	Naphto_2_6_ABz_3a4a_14	0.12	0.04	0.87	1.32	-1.08	0.12	8.40	0.00
○	■	CHEMBL476099	Naphto_2_6_ABz_2a3a_17	1.17	0.07	0.56	1.19	1.16	0.07	8.40	0.00
○	■	CHEMBL476099	Naphto_2_6_ABz_2a5a_12	1.57	0.20	1.59	1.62	1.53	0.21	8.40	0.00
○	■	CHEMBL476099	Naphto_2_6_ABz_2a6a_19	1.90	0.28	1.99	1.82	1.97	0.26	8.40	0.00
○	■	CHEMBL476099	Naphto_2_6_ABz_2a3b_6	2.13	0.33	2.21	1.94	2.32	0.29	8.40	0.00
○	■	CHEMBL476099	Naphto_2_6_ABz_3a4a_18	2.25	0.35	2.83	2.37	2.13	0.37	8.40	0.00
○	■	CHEMBL476099	Naphto_2_6_ABz_3a4b_1	2.65	0.42	3.42	2.82	2.48	0.45	8.40	0.00
○	■	CHEMBL476099	Naphto_2_6_ABz_2a2b_15	6.09	0.78	6.07	6.30	5.88	0.80	8.40	0.00
○	■	CHEMBL476099	Naphto_2_6_ABz_3a4b_3	7.22	0.85	7.18	8.95	5.50	0.95	8.40	0.00
○	■	CHEMBL476099	Naphto_2_6_ABz_2a4a_9	12.82	1.11	8.64	13.73	11.91	1.14	8.40	0.00
○	■	CHEMBL476099	Naphto_2_6_ABz_2a4b_13	30.83	1.48	11.94	37.71	23.96	1.58	8.40	0.00
○	■	CHEMBL476099	Naphto_2_6_ABz_2a5a_7	58.64	1.76	14.02	70.56	46.72	1.85	8.40	0.00
○	■	CHEMBL476099	Naphto_2_6_ABz_3a5a_11	68.25	1.81	14.81	90.69	45.81	1.96	8.40	0.00
○	■	CHEMBL476099	Naphto_2_6_ABz_2a4b_5	82.91	1.90	15.36	106.43	59.39	2.03	8.40	0.00
○	■	CHEMBL476099	Naphto_2_6_ABz_2a3b_8	1000.00	3.72	30.58	1000.00	1000.00	4.04	8.40	0.00

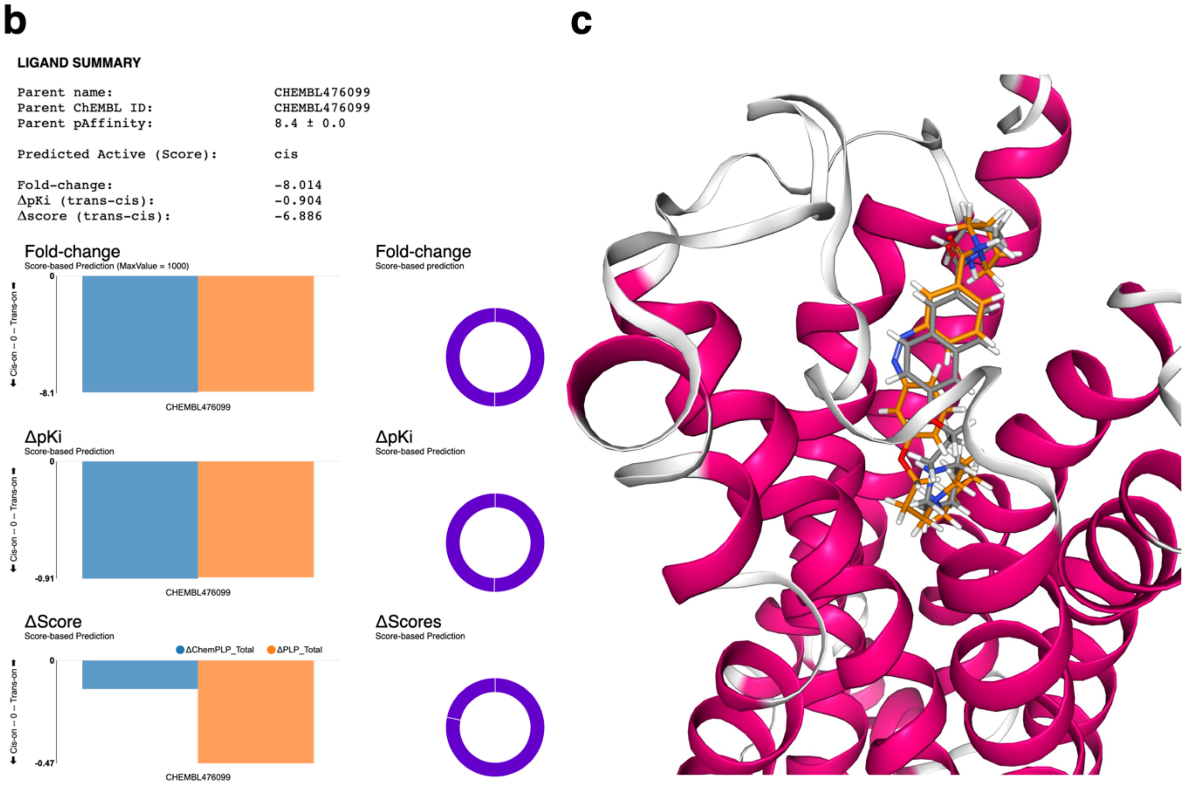

Figure S5. The interactive output of the Screening & Scoring module for photoswitchable ligands for the histamine H_3 receptor. a. Summary table filtered for the parent compound ChEMBL476099 and sorted by predicted fold-change (descending), with *cis*-ON photoswitchable ligands highlighted in purple (negative values) and *trans*-ON highlighted in green (positive values). **b.** Ligand summary, family 2D-depiction, and consensus of fold-change, ΔpK_i, and ΔScores for the ChEMBL476099 and photoswitchable analog VUF14738. **c.** Superimposition of the best-ranked docking binding pose of the parent molecule (gray) and the *cis*-VUF14738 (orange) as shown in the "Prediction" component view.

References

(1) Mendez, D.; Gaulton, A.; Bento, A. P.; Chambers, J.; De Veij, M.; Félix, E.; Magariños, M. P.; Mosquera, J. F.; Mutowo, P.; Nowotka, M.; Gordillo-Marañón, M.; Hunter, F.; Junco, L.; Mugumbate, G.; Rodriguez-Lopez, M.; Atkinson, F.; Bosc, N.; Radoux, C. J.; Segura-Cabrera, A.; Hersey, A.; Leach, A. R. ChEMBL: Towards Direct Deposition of Bioassay Data. *Nucleic Acids Res.* **2019**, *47*, D930–D940. https://doi.org/10.1093/nar/gky1075.

(2) Landrum, G.; Tosco, P.; Kelley, B.; Ric; sriniker; gedeck; Cosgrove, D.; Vianello, R.; NadineSchneider; Kawashima, E.; N, D.; Dalke, A.; Jones, G.; Cole, B.; Swain, M.; Turk, S.; AlexanderSavelyev; Vaucher, A.; Wójcikowski, M.; Take, I.; Probst, D.; Scalfani, V. F.; Ujihara, K.; godin, guillaume; Pahl, A.; Berenger, F.; JLVarjo; jasondbiggs; strets123; JP. RDKit: Open-Source Cheminformatics., 2023. https://doi.org/10.5281/zenodo.7671152.

(3) Yung-Chi, C.; Prusoff, W. H. Relationship between the Inhibition Constant (KI) and the Concentration of Inhibitor Which Causes 50 per Cent Inhibition (I50) of an Enzymatic Reaction. *Biochem. Pharmacol.* **1973**, *22*, 3099–3108. https://doi.org/10.1016/0006-2952(73)90196-2.

(4) Hüll, K.; Morstein, J.; Trauner, D. In VivoPhotopharmacology. *Chem. Rev.* **2018**, *118*, 10710–10747. https://doi.org/10.1021/acs.chemrev.8b00037.

(5) Wijtmans, M.; Josimovic, I.; Vischer, H. F.; Leurs, R. Optical Control of Class A G Protein-Coupled Receptors with Photoswitchable Ligands. *Curr. Opin. Pharmacol.* **2022**, *63*, 102192. https://doi.org/10.1016/j.coph.2022.102192.

(6) Kobauri, P.; Szymanski, W.; Cao, F.; Thallmair, S.; Marrink, S. J.; Witte, M. D.; Dekker, F. J.; Feringa, B. L. Biaryl Sulfonamides as Cisoid Azosteres for Photopharmacology. *Chem. Commun.* **2021**, *57*, 4126–4129. https://doi.org/10.1039/D1CC00950H.

(7) Halgren, T. A. Merck Molecular Force Field. I. Basis, Form, Scope, Parameterization, and Performance of MMFF94. *J. Comput. Chem.* **1996**, *17*, 490–519. https://doi.org/10.1002/(SICI)1096-987X(199604)17:5/6<490::AID-JCC1>3.0.CO;2-P.

(8) O'Boyle, N. M.; Banck, M.; James, C. A.; Morley, C.; Vandermeersch, T.; Hutchison, G. R. Open Babel: An Open Chemical Toolbox. *J. Cheminformatics* **2011**, *3*, 33. https://doi.org/10.1186/1758-2946-3-33.

(9) Korb, O.; Stützle, T.; Exner, T. E. An Ant Colony Optimization Approach to Flexible Protein–Ligand Docking. *Swarm Intell.* **2007**, *1*, 115–134. https://doi.org/10.1007/s11721-007-0006-9.

(10) UniProt Consortium. UniProt: A Hub for Protein Information. *Nucleic Acids Res.* **2015**, *43*, D204–D212. https://doi.org/10.1093/nar/gku989.

(11) Burley, S. K.; Bhikadiya, C.; Bi, C.; Bittrich, S.; Chen, L.; Crichlow, G. V.; Christie, C. H.; Dalenberg, K.; Di Costanzo, L.; Duarte, J. M.; Dutta, S.; Feng, Z.; Ganesan, S.; Goodsell, D. S.; Ghosh, S.; Green, R. K.; Guranović, V.; Guzenko, D.; Hudson, B. P.; Lawson, C. L.; Liang, Y.; Lowe, R.; Namkoong, H.; Peisach, E.; Persikova, I.; Randle, C.; Rose, A.; Rose, Y.; Sali, A.; Segura, J.; Sekharan, M.; Shao, C.; Tao, Y.-P.; Voigt, M.; Westbrook, J. D.; Young, J. Y.; Zardecki, C.; Zhuravleva, M. RCSB Protein Data Bank: Powerful New Tools for Exploring 3D Structures of Biological Macromolecules for Basic and Applied Research and Education in Fundamental Biology,

Biomedicine, Biotechnology, Bioengineering and Energy Sciences. *Nucleic Acids Res.* **2021**, *49*, D437–D451. https://doi.org/10.1093/nar/gkaa1038.

(12)Berman, H. M.; Westbrook, J.; Feng, Z.; Gilliland, G.; Bhat, T. N.; Weissig, H.; Shindyalov, I. N.; Bourne, P. E. The Protein Data Bank. *Nucleic Acids Res* **2000**, *28*, 235–242. https://doi.org/10.1093/nar/28.1.235.

(13)Roughley, S. D. Five Years of the KNIME Vernalis Cheminformatics Community Contribution. *Curr. Med. Chem. 27*, 6495–6522.

(14)McGuire, R.; Verhoeven, S.; Vass, M.; Vriend, G.; de Esch, I. J. P.; Lusher, S. J.; Leurs, R.; Ridder, L.; Kooistra, A. J.; Ritschel, T.; de Graaf, C. 3D-e-Chem-VM: Structural Cheminformatics Research Infrastructure in a Freely Available Virtual Machine. *J. Chem. Inf. Model.* **2017**, *57*, 115–121. https://doi.org/10.1021/acs.jcim.6b00686.

(15)Jumper, J.; Evans, R.; Pritzel, A.; Green, T.; Figurnov, M.; Ronneberger, O.; Tunyasuvunakool, K.; Bates, R.; Žídek, A.; Potapenko, A.; Bridgland, A.; Meyer, C.; Kohl, S. A. A.; Ballard, A. J.; Cowie, A.; Romera-Paredes, B.; Nikolov, S.; Jain, R.; Adler, J.; Back, T.; Petersen, S.; Reiman, D.; Clancy, E.; Zielinski, M.; Steinegger, M.; Pacholska, M.; Berghammer, T.; Bodenstein, S.; Silver, D.; Vinyals, O.; Senior, A. W.; Kavukcuoglu, K.; Kohli, P.; Hassabis, D. Highly Accurate Protein Structure Prediction with AlphaFold. *Nature* **2021**, *596*, 583–589. https://doi.org/10.1038/s41586-021-03819-2.

(16)Korb, O.; Stützle, T.; Exner, T. E. Empirical Scoring Functions for Advanced Protein-Ligand Docking with PLANTS. *J. Chem. Inf. Model.* **2009**, *49*, 84–96. https://doi.org/10.1021/ci800298z.

(17)PDBFixer, 2023. https://github.com/openmm/pdbfixer (accessed 2023-02-17).

(18)Dolinsky, T. J.; Czodrowski, P.; Li, H.; Nielsen, J. E.; Jensen, J. H.; Klebe, G.; Baker, N. A. PDB2PQR: Expanding and Upgrading Automated Preparation of Biomolecular Structures for Molecular Simulations. *Nucleic Acids Res.* **2007**, *35*, W522–W525. https://doi.org/10.1093/nar/gkm276.

(19)Olsson, M. H. M.; Søndergaard, C. R.; Rostkowski, M.; Jensen, J. H. PROPKA3: Consistent Treatment of Internal and Surface Residues in Empirical pK_a Predictions. *J. Chem. Theory Comput.* **2011**, *7*, 525–537. https://doi.org/10.1021/ct100578z.

(20)Vanommeslaeghe, K.; Hatcher, E.; Acharya, C.; Kundu, S.; Zhong, S.; Shim, J.; Darian, E.; Guvench, O.; Lopes, P.; Vorobyov, I.; Mackerell Jr., A. D. CHARMM General Force Field: A Force Field for Drug-like Molecules Compatible with the CHARMM All-Atom Additive Biological Force Fields. *J. Comput. Chem.* **2010**, *31*, 671–690. https://doi.org/10.1002/jcc.21367.
